# Supplementary material for: Hepatocyte FBXW7-dependent activity of nutrient-sensing nuclear receptors controls systemic energy homeostasis and NASH progression in male mice
Source: Nat Commun. 2023 Nov 1;14:6982. doi: 10.1038/s41467-023-42785-3 (PMC10620240; doi:10.1038/s41467-023-42785-3)
Supplement: Supplementary file 1 — Supplementary Information [file 41467_2023_42785_MOESM1_ESM.pdf]

Hepatocyte FBXW7-dependent activity of nutrient-sensing nuclear receptors controls systemic energy homeostasis and NASH progression in male mice

Hui Xia[1,2], Catherine R. Dufour[1], Younes Medkour[1], Charlotte Scholtes[1], Yonghong Chen[1,2], Christina Guluzian[1,2], Wafa B'chir[1], and Vincent Giguère[1,2]

[1]Goodman Cancer Institute, McGill University, Montréal, Québec, Canada H3A 1A3.

[2]Department of Biochemistry, Faculty of Medicine and Health Sciences, McGill University, Montréal, Québec, Canada H3G 1Y6.

✉e-mail **:** vincent.giguere@mcgill.ca (V.G.)

Supplemental Information

Supplementary Fig. 1-9

Supplementary Data 1-8 (excel files)

Supplementary Fig. 1

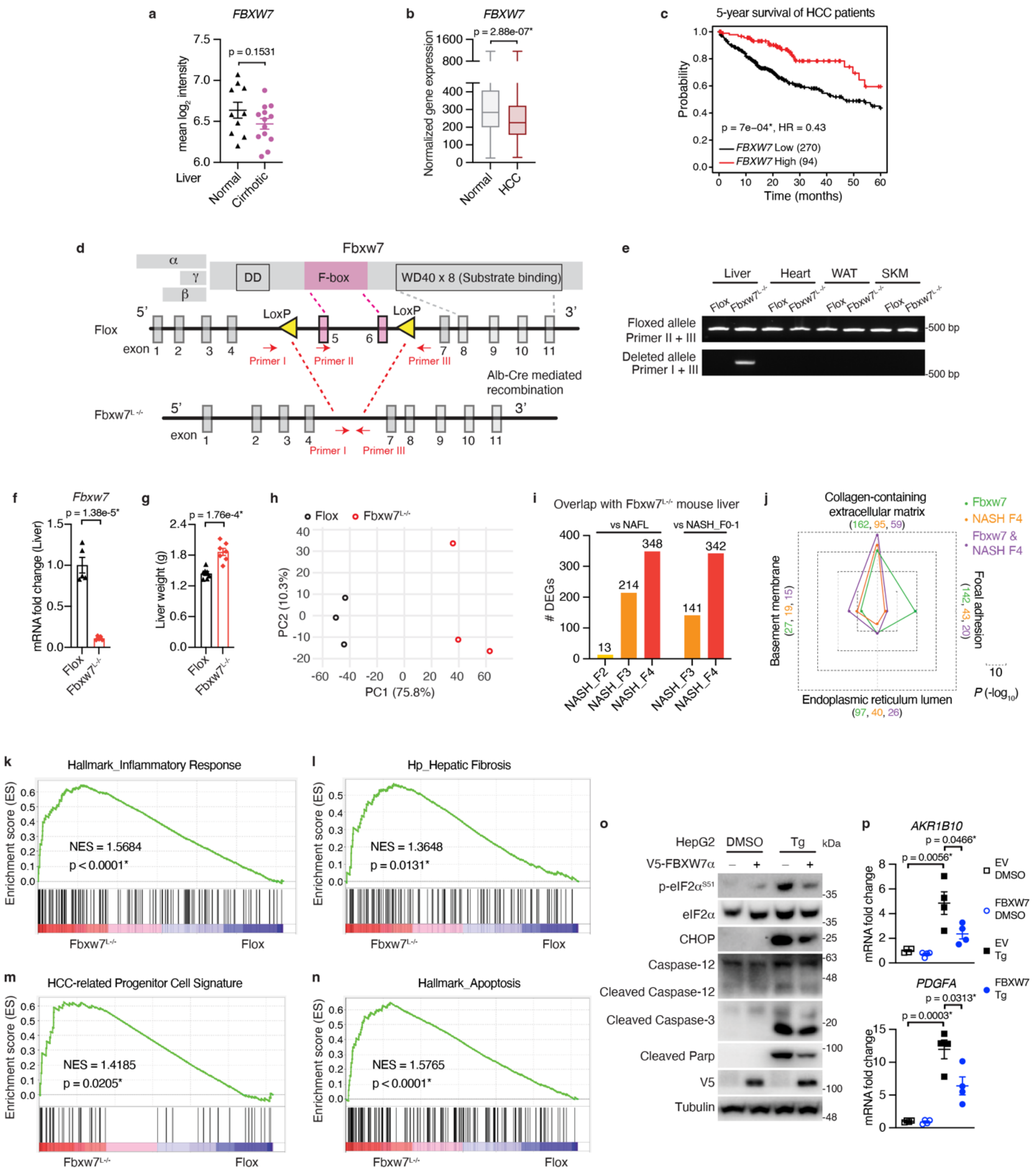

Supplementary Fig. 1 | FBXW7 protects against NASH. a *FBXW7* mRNA levels in normal (n = 10) and cirrhotic (n = 13) human livers. **b** Normalized *FBXW7* gene expression levels in normal liver (n = 225) and hepatocellular carcinoma (HCC, n = 371). Box plots show centre line

as median, bounds of box as 25th and 75th percentiles, whiskers as minima and maxima. **c** Effects of high (n = 94) and low (n = 270) *FBXW7* expression on 5-year survival rate of patients with HCC (n = 364). HR, hazard ratio. **d** Schematic diagram of the Fbxw7 gene and protein as well as stratagem used for liver-specific deletion of its F-box domain required for ubiquitination complex assembly. Exons corresponding to the F-box domain and the loxP sites are color-coded. Arrows indicate locations of primers (I–III) used for genotyping. **e** Liver-specific excision of exons 5 and 6 upon Alb-Cre mediated recombination, revealed by PCR. **f** RT-qPCR analyses of liver *Fbxw7* transcript levels using primers located in exons 3 and 5, n = 5. **g** Liver weights of *Flox* and $Fbxw7^{L-/-}$ mice, n = 7. **h** Principal component analysis (PCA) plot of liver RNA-seq replicates of *Flox* and $Fbxw7^{L-/-}$ mice, n = 3. **i** Overlap of DEGs in Fbxw7-null mouse livers ($p < 0.05$, $|FC| > 1.30$) and human NASH progression signatures using either NAFL or NASH F0/F1 as baseline (FDR < 0.05). **j** GO cellular component analysis of 342 DEGs in (**i**) shared in Fbxw7-null livers and NASH F4 (using NASH F0/F1 as baseline). A –log10 (p-value) was used to present the top enriched terms. The number of genes per category are indicated in parentheses. **k-n** GSEA of the indicated signatures in $Fbxw7^{L-/-}$ and *Flox* mice. Array genes were ordered from the highest in Fbxw7-null livers (left) to the highest in control livers (right). Locations of genes in the signature are indicated by the vertical black bars. NES: normalized enrichment score. **o**, **p** HepG2 cells stably expressing empty vector (EV) or FBXW7 were treated with 500 nM Thapsigargin (Tg) or DMSO for 24 h. Markers of ER stress and apoptosis as well as NASH progression genes were examined using immunoblots (**o**) and RT-qPCR (**p**), respectively. Data are represented as means ± SEM (**a**, **f, g, p**). *$p < 0.05$, unpaired two-tailed Student's t test (**a**, **f, g, p**), Mann-Whitney U test (**b**), Kaplan–Meier method (**c**). Source data are provided as a Source Data file.

Supplementary Fig. 2

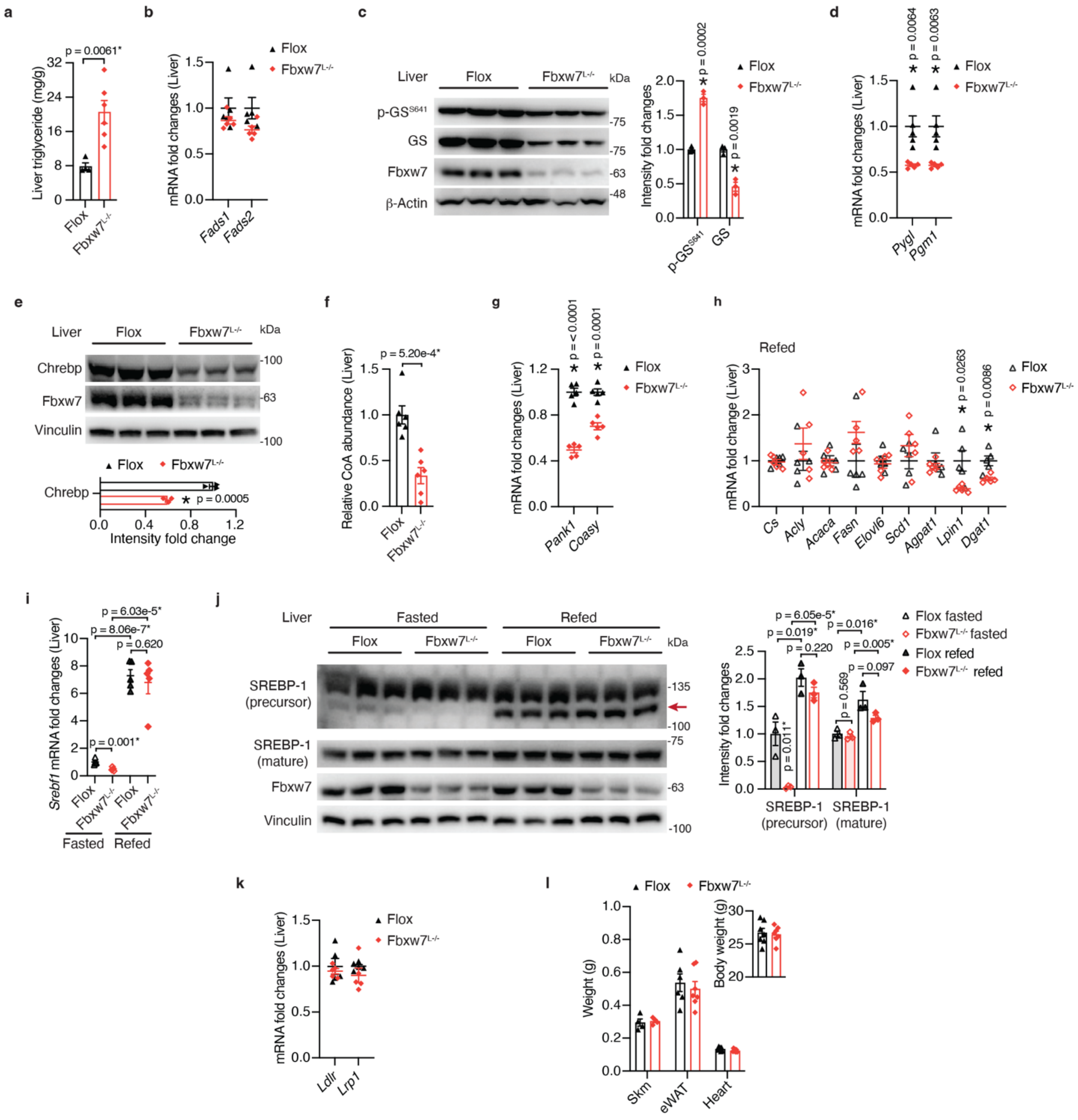

Supplementary Fig. 2 | Fat accumulation in Fbxw7-null livers is independent of hepatic lipogenesis. a Liver triglyceride contents of fed *Flox* and *Fbxw7*$^{L-/-}$ littermates, n = 4-6. **b** Liver mRNA levels of *Fads1* and *Fads2* from *Flox* and *Fbxw7*$^{L-/-}$ littermates, n = 5. **c** Immunoblots and quantification of hepatic proteins in glycogen metabolism. Each lane represents one mouse, n =

3. **d** Liver mRNA expression of glycogenolysis genes from *Flox* and *Fbxw7*$^{L-/-}$ littermates, n = 5. **e** Immunoblots and quantification of hepatic Chrebp protein. Each lane represents one mouse, n = 3. **f** Hepatic CoA abundance, n = 6. **g** Liver mRNA levels of genes involved in CoA biosynthesis, n = 5. **h** Liver mRNA expression of lipogenic genes of *Flox* and *Fbxw7*$^{L-/-}$ littermates post 2 h-refeeding following a 22 h starvation, n = 5. **i** Hepatic *Srebf1* mRNA levels during the fasting and refeeding transition, n = 5. **j** Immunoblots and quantification of hepatic SREBP-1 protein during the fasting and refeeding transition, precursor SREBP-1 protein was denoted with red arrow. Each lane represents one mouse, n = 3. **k** mRNA expression of genes involved in hepatic uptake of chylomicron remnants, n = 5. **l** body weight (n = 7) and tissue weights (n = 4-7) of *Flox* and *Fbxw7*$^{L-/-}$ mice. Data are represented as means ± SEM, *p < 0.05, unpaired two-tailed Student's t test (**a-l**). Source data are provided as a Source Data file.

Supplementary Fig. 3

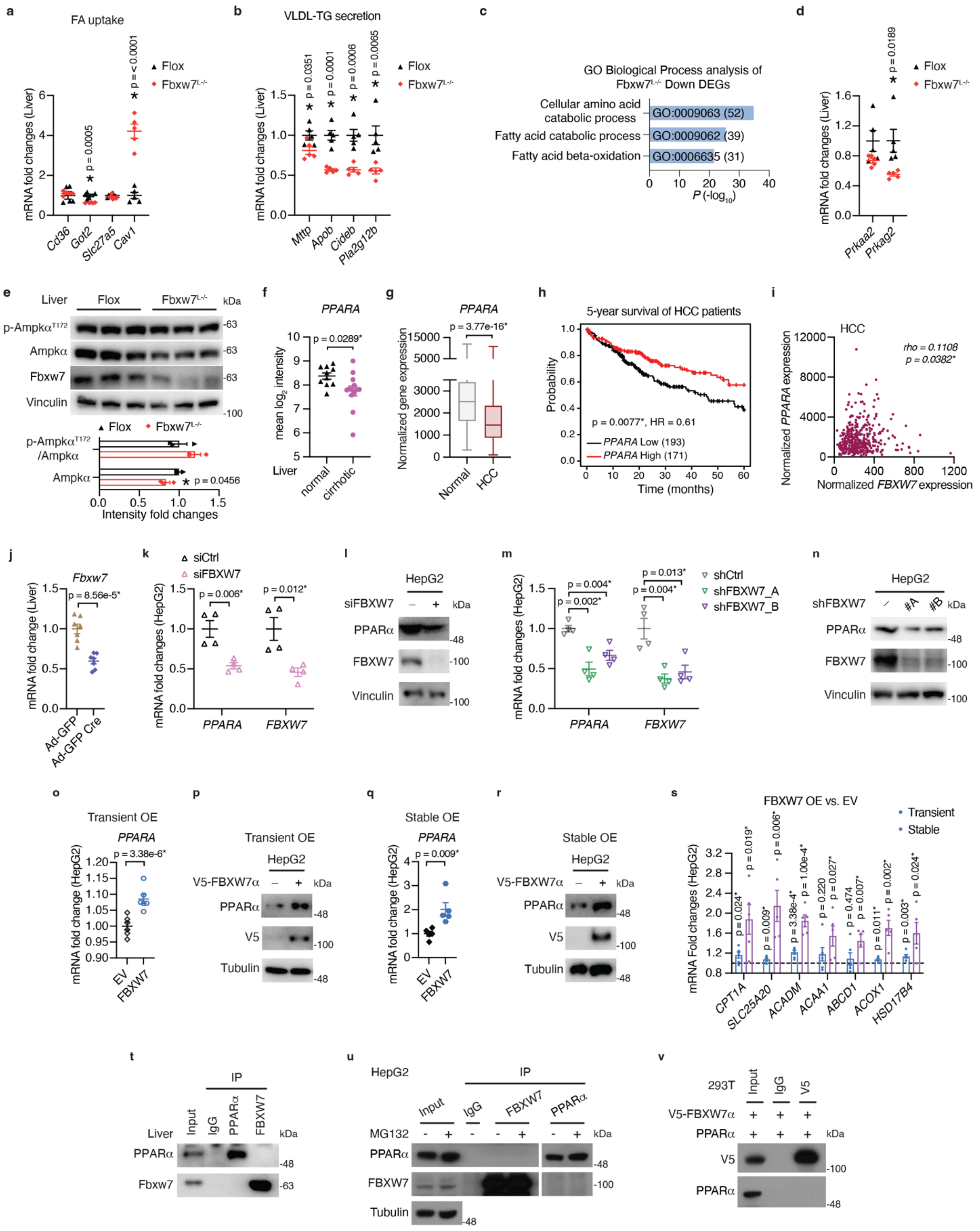

Supplementary Fig. 3 | Loss of Fbxw7 impairs PPARα-dependent lipid oxidation. a, **b** RT-qPCR analysis of genes involved in hepatic FA uptake (**a**) and VLDL-TG secretion (**b**) of *Flox* and *Fbxw7$^{L-/-}$* littermates, n = 5. **c** GO biological process analysis of DEGs ($p < 0.05$, $|FC| > 1.30$) in Fbxw7-null livers. Shown are the top three enriched terms. The number of genes in each category are shown in parentheses. **d** RT-qPCR analysis of Ampk subunits in control and Fbxw7-null livers, n = 5. **e** Immunoblots and quantification of phosphorylated and total Ampkα proteins in control and Fbxw7-null livers. Each lane represents one mouse, n = 3. **f** *PPARA* mRNA levels in normal (n = 10) and cirrhotic (n = 13) human livers. **g** Normalized *PPARA* gene expression levels in normal liver (n = 225) and hepatocellular carcinoma (HCC, n = 371). Box plots show centre line as median, bounds of box as 25th and 75th percentiles, whiskers as minima and maxima. **h** Effects of high (n = 171) and low (n = 193) *PPARA* expression on 5-year survival rate of patients with HCC (n = 364). HR, hazard ratio. **i** Correlation analysis of *FBXW7* mRNA level with *PPARA* in HCC, n = 371. **j** Hepatic *Fbxw7* mRNA level in Fbxw7 *Flox* mice injected with GFP or GFP-Cre adenovirus, n = 7. **k**, **l** mRNA (**k**, n = 4) and protein (**l**) levels of PPARα and FBXW7 in HepG2 cells transfected with 20 nM of either control or FBXW7 siRNA for 72 h. **m**, **n** mRNA (**m**, n = 4) and protein (**n**) levels of PPARα and FBXW7 in HepG2 cells stably expressing either control shRNA or 2 distinct shRNAs targeting FBXW7. **o**, **p** mRNA (**o**, n = 6) and protein (**p**) levels of PPARα in HepG2 cells transfected with FBXW7 or empty vector (EV) for 48 h. **q**, **r** mRNA (**q**, n = 5) and protein (**r**) levels of PPARα in HepG2 cells stably overexpressing (OE) FBXW7 or EV. **s** RT-qPCR analysis of FAO genes in HepG2 cells transiently (n = 6) and stably (n = 5) overexpressing FBXW7 relative to EV. **t, u, v** Immunoprecipitation studies performed using liver lysates equally pooled from three mice (**t**), HepG2 cells treated with DMSO or 20 μM MG132 for 6 h (**u**), and 293T cells transiently co-

expressing V5-FBXW7 and PPARα vector for 48 h (**v**). Data are represented as means ± SEM (**a**, **b**, **d-f**, **j**, **k**, **m**, **o**, **q**, **s**). *$p < 0.05$, unpaired two-tailed Student's t test (**a**, **b**, **d-f**, **j**, **k**, **m**, **o**, **q**, **s**), Mann-Whitney U test (**g**), Kaplan–Meier method (**h**), non-parametric Spearman's test (**i**). Source data are provided as a Source Data file.

Supplementary Fig. 4

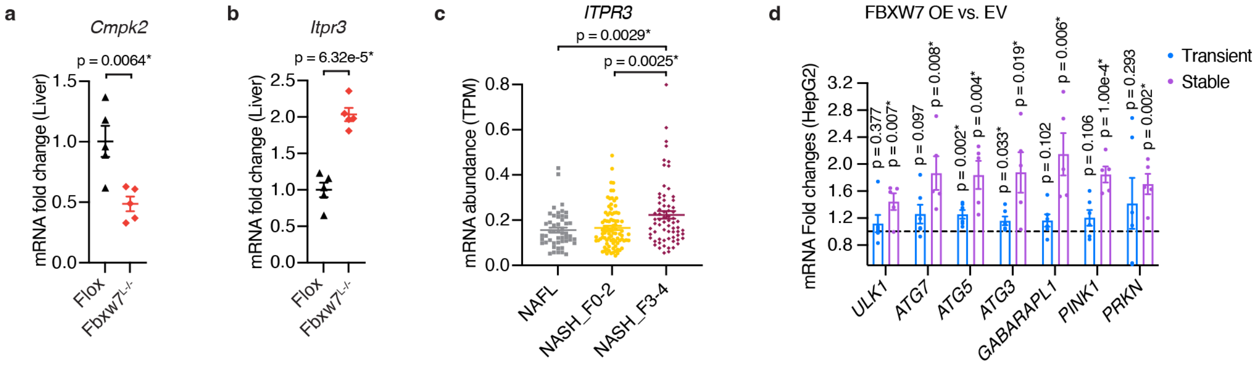

Supplementary Fig. 4 | Fbxw7-null livers exhibit mitochondrial dysfunction and autophagy deficiency. a, **b** mRNA expression of *Cmpk2* (**a**) and *Itpr3* (**b**) in control and Fbxw7-null livers, n = 5. **c** Analysis of hepatic *ITPR3* expression in patients across the severity stages of NAFLD progression. n = 50 for NAFL; n = 87 for NASH_F0-2; n = 68 for NASH_F3-4. **d** RT-qPCR analysis of autophagy genes in HepG2 cells transiently (n = 6) and stably (n = 5) overexpressing FBXW7 relative to empty vector (EV). Data are represented as means ± SEM, *p < 0.05, unpaired two-tailed Student's t test (**a-d**). Source data are provided as a Source Data file.

Supplementary Fig. 5

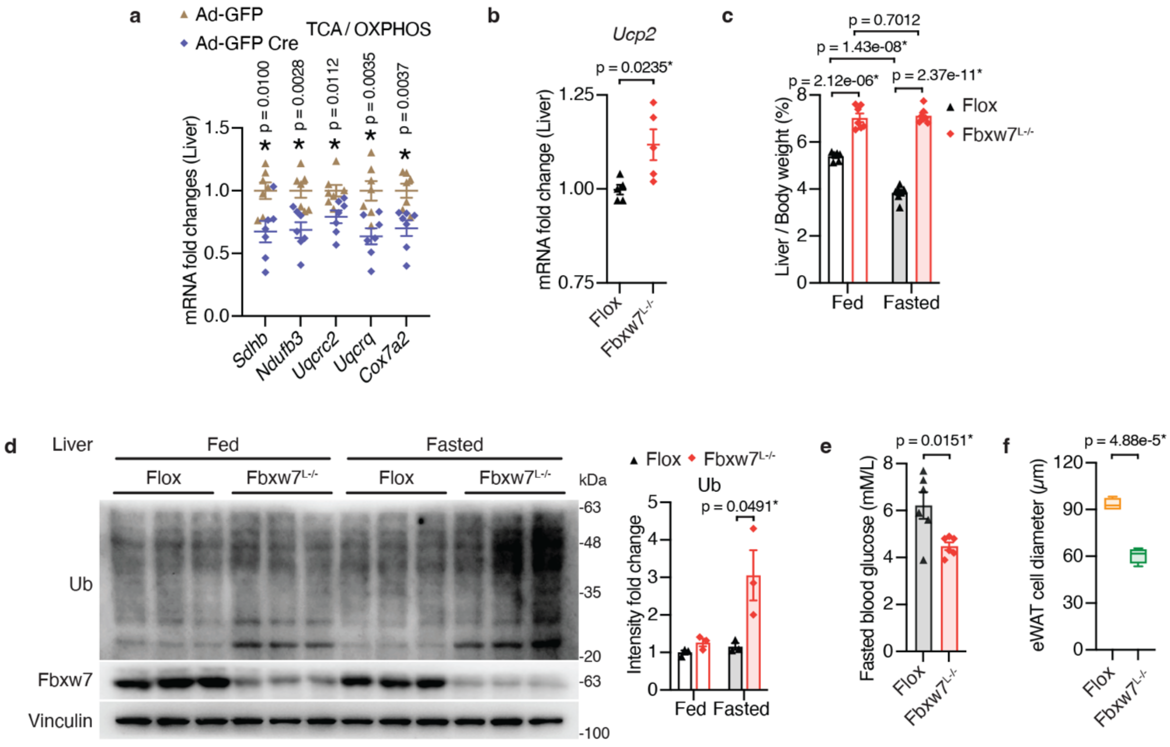

Supplementary Fig. 5 | *Fbxw7$^{L-/-}$* mice adapt poorly to starvation. a Hepatic mRNA expression of TCA cycle and OXPHOS genes of Fbxw7 *Flox* mice injected with adenovirus expressing GFP or Cre recombinase, n = 7. **b** mRNA expression of *Ucp2* in control and Fbxw7-null livers, n = 5. **c** Liver to body weight (%) of fed and fasted *Flox* and *Fbxw7$^{L-/-}$* mice, n = 7-8. **d** Immunoblots and quantification of polyubiquitinated proteins from *Flox* and Fbxw7$^{L-/-}$ mice in the fed and fasted states. Each lane represents one mouse, n = 3. **e** Levels of fasted blood glucose in *Flox* and *Fbxw7$^{L-/-}$* mice, n = 6. **f** Quantitation of eWAT adipocyte sizes, n = 4. Four random sections per mouse were quantified. Box plots show centre line as median, bounds of box as 25th and 75th percentiles, whiskers as minima and maxima. See Fig. 5**m** for the representative stained sections. Data are represented as means ± SEM (**a**-**e**). *$p < 0.05$, unpaired two-tailed Student's t test (**a**-**f**). Source data are provided as a Source Data file.

Supplementary Fig. 6

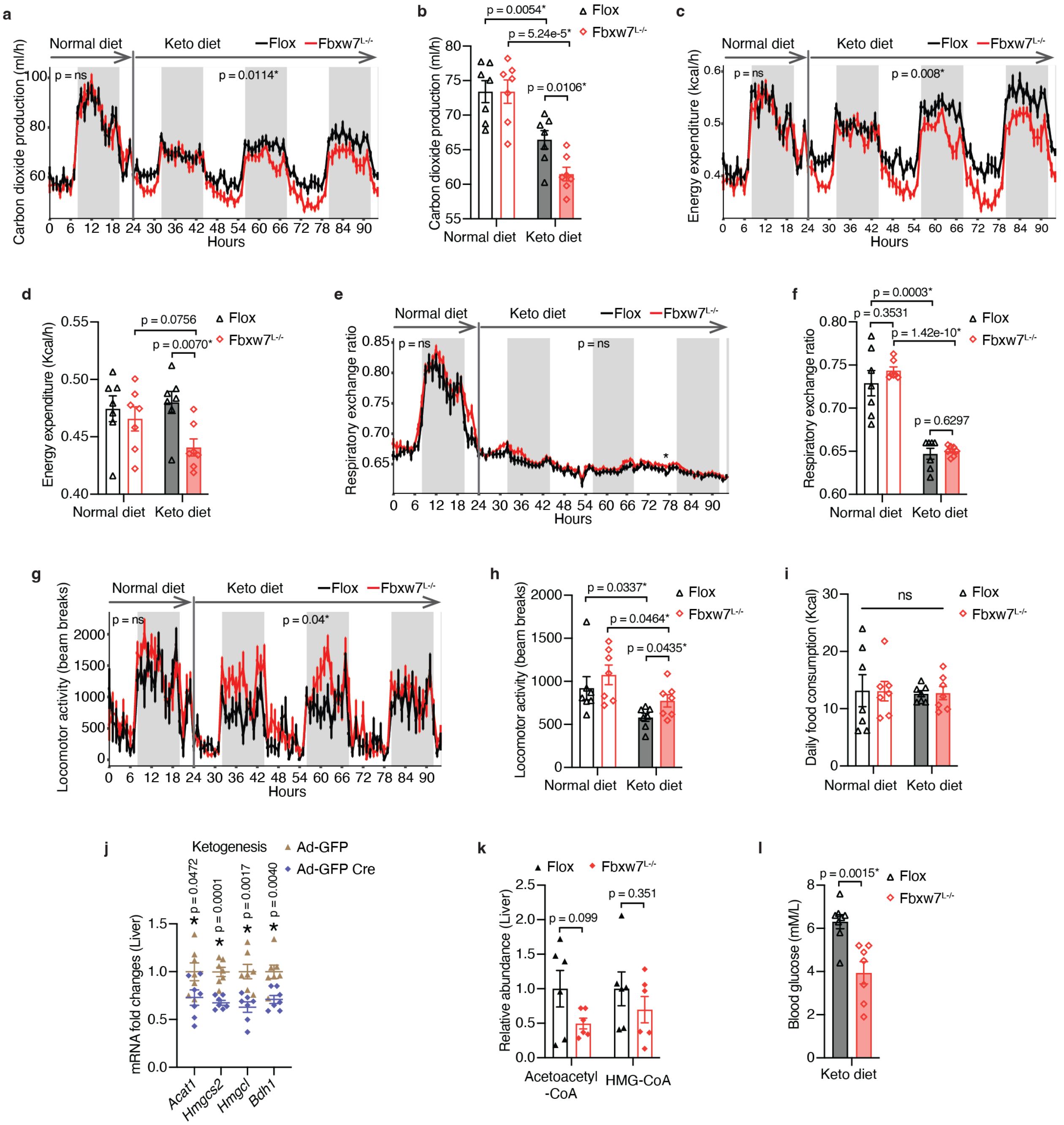

Supplementary Fig. 6 | Hepatic Fbxw7 deficiency inhibits ketogenic diet-induced fat utilization. a, **b** Hourly plot (**a**) and overall average (**b**) of whole-body carbon dioxide production of *Flox* and *Fbxw7*$^{L-/-}$ mice switching from a normal diet to keto diet, n = 7. **c**, **d** Hourly plot (**c**) and overall average (**d**) of whole-body energy expenditure of *Flox* and *Fbxw7*$^{L-/-}$

mice under a normal or keto diet, n = 7. **e**, **f** Hourly plot (**e**) and overall average (**f**) of respiratory exchange ratio of *Flox* and $Fbxw7^{L-/-}$ mice upon switching from a normal diet to keto diet, n = 7. **g**, **h** Hourly plot (**g**) and overall average (**h**) of locomotor activity of *Flox* and $Fbxw7^{L-/-}$ mice under a normal or keto diet, n = 7. **i** Daily food consumption of *Flox* and $Fbxw7^{L-/-}$ mice switching from a normal diet to keto diet, n = 7. **j** Hepatic mRNA expression of ketogenesis genes of Fbxw7 *Flox* mice injected with adenovirus expressing GFP or Cre recombinase, n = 7. **k** Relative abundance of fatty acyl-CoAs involved in ketogenesis from *Flox* and Fbxw7-null livers, n = 6. **l** Blood glucose concentrations of *Flox* and $Fbxw7^{L-/-}$ mice post a 20-day keto diet, n = 7-8. Data are represented as means ± SEM (**a-l**). *$p < 0.05$, unpaired two-tailed Student's t test (**b**, **d**, **f**, **h-l**), one-way ANCOVA using body weight as the covariate (**a**, **c**), and one-way ANOVA (**e**, **g**). Source data are provided as a Source Data file.

Supplementary Fig. 7

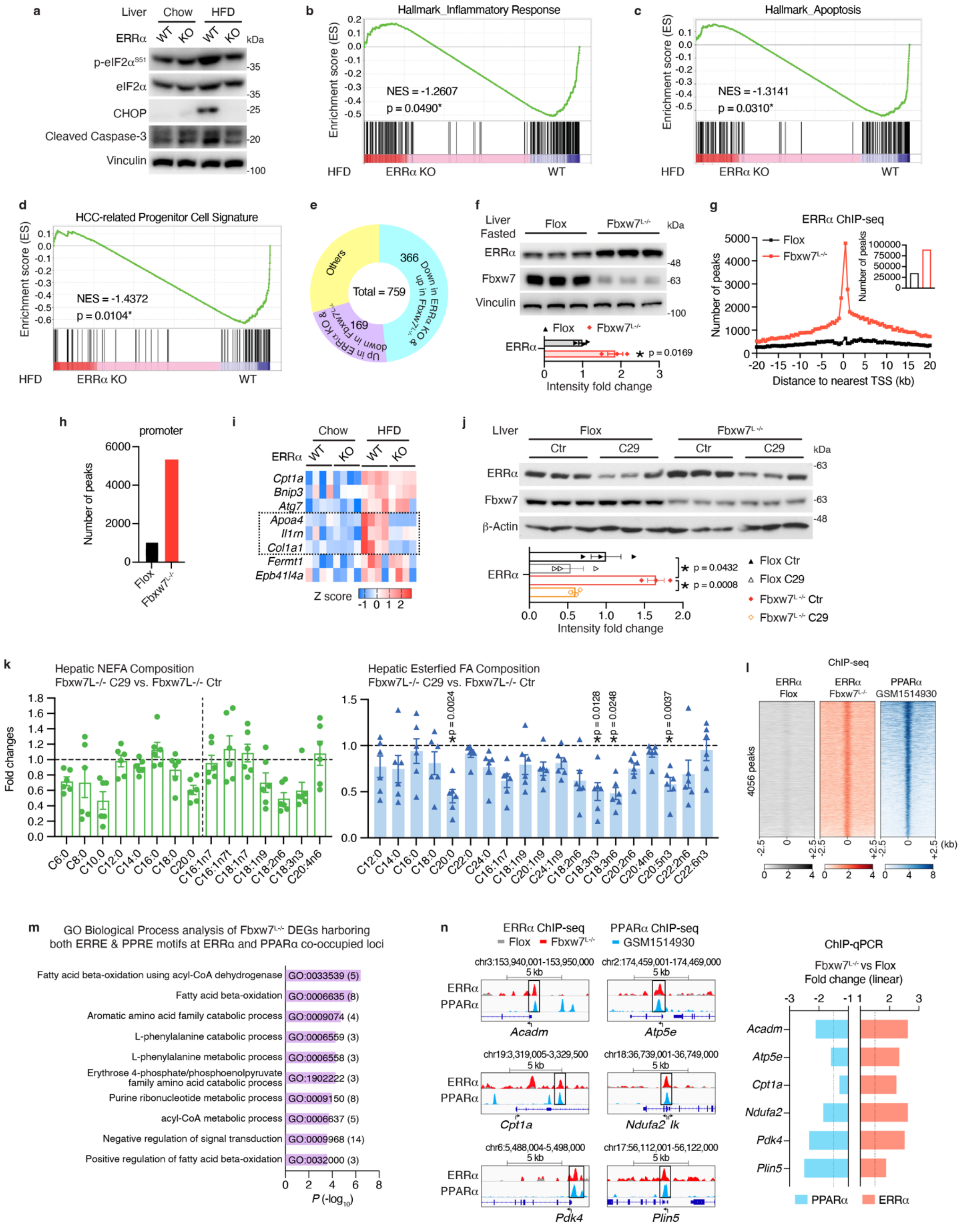

Supplementary Fig. 7 | Inhibition of Fbxw7 substrate ERRα alleviates NASH. a Immunoblots of hepatic markers of ER stress and apoptosis from ERRα WT and KO mice fed a chow or a HFD for 15 weeks. Each lane represents a sample equally pooled from four mice. **b**, **c**, **d** GSEA of the indicated signatures in HFD-fed ERRα KO mice and WT controls. Array genes were ordered from the highest in livers of ERRα KO mice (left) to the highest in livers of WT mice (right). Locations of genes in each signature are indicated by the vertical black bars. **e** Overlap of DEGs identified in livers from HFD-fed ERRα-null and *Fbxw7*$^{L-/-}$ mice ($p < 0.05$, $|FC| > 1.30$). **f** Immunoblots and quantification of hepatic ERRα proteins from fasted *Flox* and *Fbxw7*$^{L-/-}$ mice. Each lane represents one mouse, n = 3. **g** Distribution of ERRα ChIP-seq peaks ± 20 kb relative to the TSS of the nearest gene identified in fasted *Flox* and *Fbxw7*$^{L-/-}$ mice. Inner histogram shows the total number of peaks bound by ERRα within ±20 kb of TSS. **h** Number of promoter-annotated ERRα ChIP-seq peaks. **i** Z score heatmap of the indicated genes in livers from WT and ERRα KO mice under a chow diet or a HFD, n = 4. Genes bordered with light-dotted lines are significantly induced by a HFD in WT but not ERRα KO mice. **j** Immunoblots and quantification of hepatic ERRα protein levels from *Flox* and *Fbxw7*$^{L-/-}$ littermates post control or C29 injection. Each lane represents one mouse, n = 3. **k** Relative composition of hepatic FFAs and esterified FAs in *Fbxw7*$^{L-/-}$ mice treated with C29 versus control, n = 6 for FFA, n = 6-7 for esterified FA. See also Supplementary Data 3. **l** Heatmaps of liver ChIP-seq read densities for ERRα from fasted control and Fbxw7-null mice as well as PPARα from GW7647-treated WT mice (GSM1514930). Read densities are shown in a window of ±2.5kb from peak summits centered at 0. **m** GO biological process analysis of a subset of shared PPARα and ERRα (*Fbxw7*$^{L-/-}$) ChIP-seq target genes (±20 kb) harboring PPRE and ERRE motifs in overlapping peaks (Fig. 8**j**) also found differentially regulated in Fbxw7-null livers ($p < 0.05$,

|FC| > 1.30). The number of genes in each category are shown in parentheses. **n** ERRα ChIP-seq tracks in control and Fbxw7-null livers and PPARα ChIP-seq in GW7647-treated livers (GSM1514930) for selected co-targeted loci identified in Fig. 8**j**. Relative ChIP-qPCR binding fold changes in Fbxw7-null versus control liver for both ERRα and PPARα at the indicated co-targeted loci. ChIP-qPCR assays for each genotype were performed on the same chromatin batch from a pool of two mice performed in single replicate. Data are represented as means ± SEM, $^*p < 0.05$, unpaired two-tailed Student's t test (**f**, **j, k**). Source data are provided as a Source Data file.

Supplementary Fig. 8

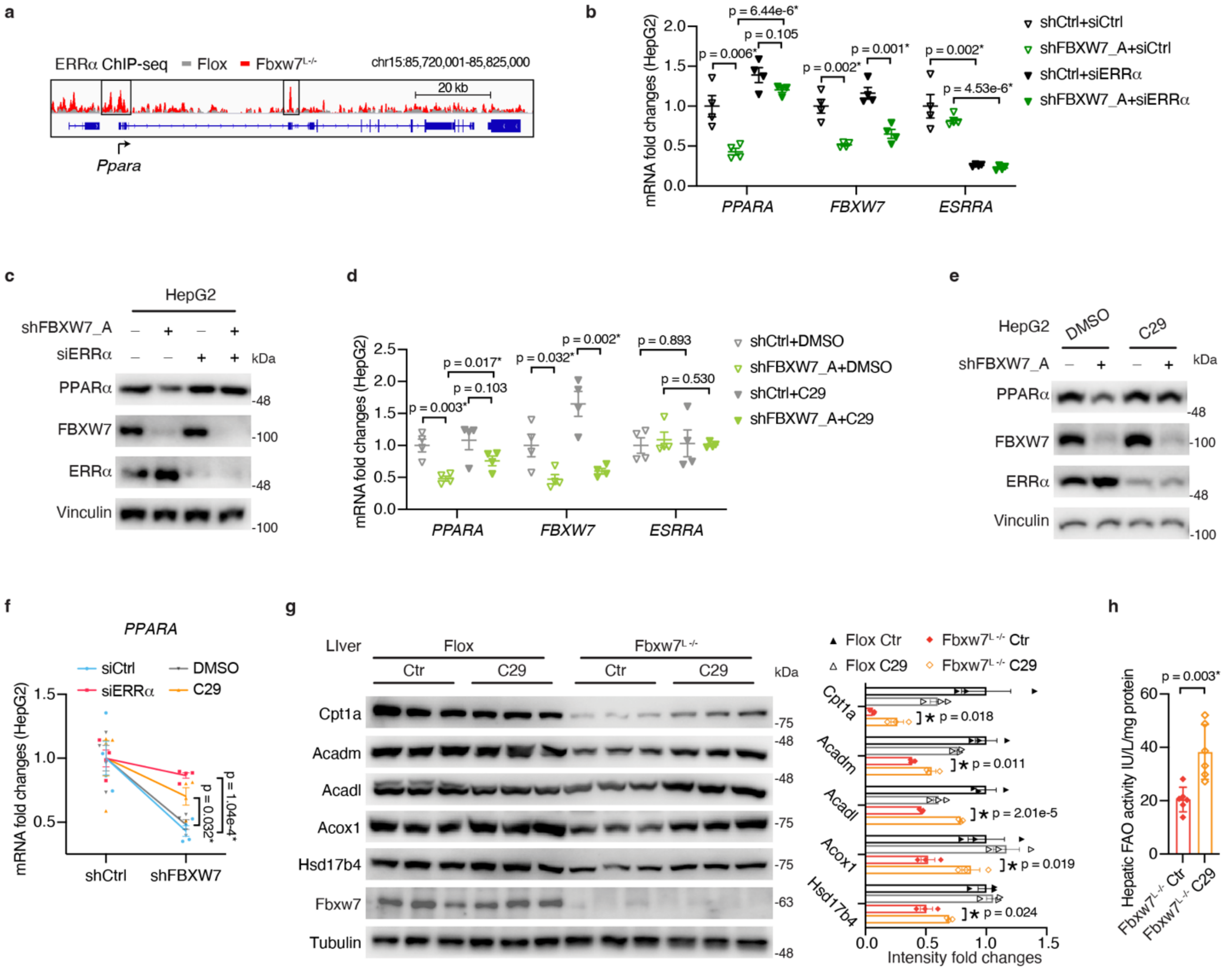

Supplementary Fig. 8 | Transcriptional down-regulation of PPARα upon Fbxw7 loss is ERRα-dependent. **a** ERRα ChIP-seq tracks in control and Fbxw7-null livers at the *Ppara* locus. Genomic regions highlighted by a blue box denote notable increased recruitment of ERRα. **b**, **c** HepG2 cells stably expressing control shRNA or shFBXW7 were transfected with 20 nM of either control or ERRα siRNA for 72 h and subjected to RT-qPCR (**b**, n = 4) and immunoblot (**c**) analyses. **d**, **e** HepG2 cells stably expressing control shRNA or shFBXW7 were treated with either DMSO or 5 µM C29 for 24 h followed by RT-qPCR (**d**, n = 4) and immunoblot (**e**) analyses. **f** *PPARA* repression induced by FBXW7 depletion in HepG2 cells were attenuated by

ERRα inhibition, n = 4. **g** Immunoblots and quantification of hepatic FAO proteins from *Flox* and *Fbxw7*$^{L-/-}$ littermates post control or C29 injection. Each lane represents one mouse, n = 3. **h** Hepatic FAO activities in *Fbxw7*$^{L-/-}$ mice post control or C29 injection, n = 6. Data are represented as means ± SEM, *p < 0.05, unpaired two-tailed Student's t test (**b**, **d**, **f**-**h**). Source data are provided as a Source Data file.

Supplementary Fig. 9

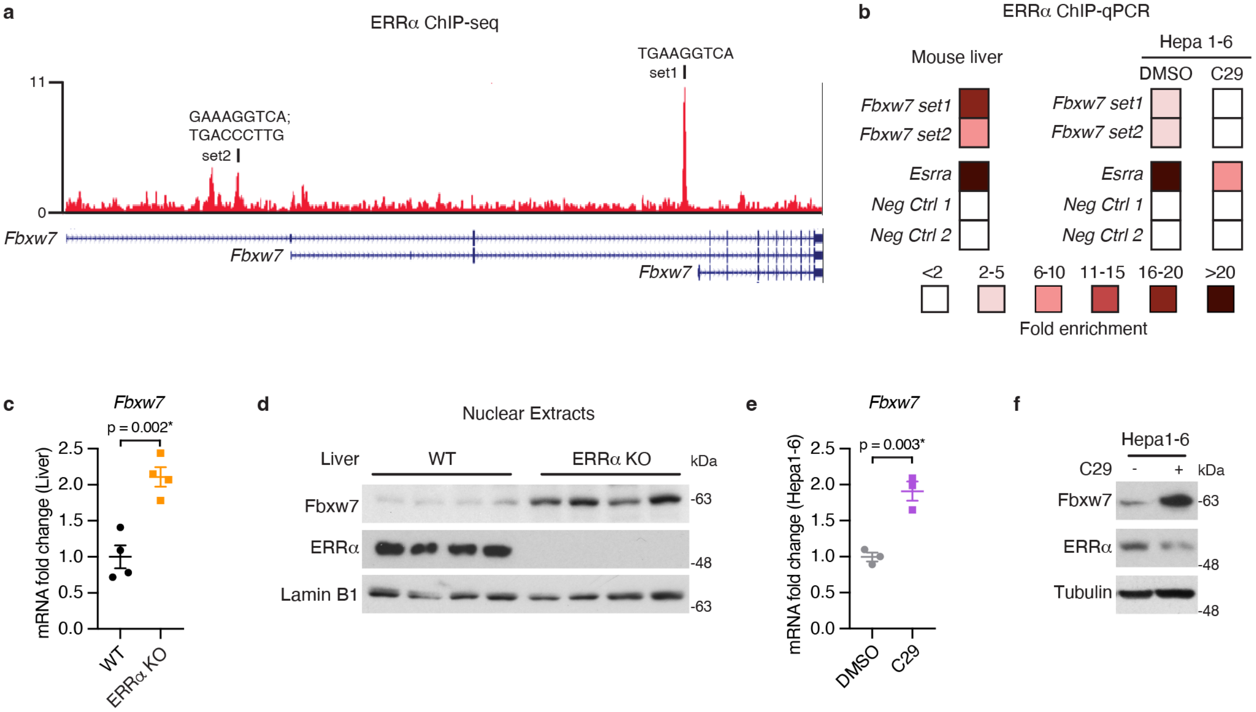

Supplementary Fig. 9 | ERRα transcriptionally represses *Fbxw7*. a ERRα ChIP-seq track for *Fbxw7* in the liver. Putative ERRα binding sequences (ERREs) are shown at the indicated binding peaks. **b** ChIP-qPCR analysis of ERRα binding to *Esrra* or *Fbxw7* in mouse liver (left) or Hepa 1-6 cells treated with DMSO or 5 μM C29 for 24 h (right). **c** Hepatic mRNA expression of *Fbxw7* in WT and ERRα KO mice, n = 4. **d** Nuclear Fbxw7 protein levels in livers from WT and ERRα KO mice. Each lane represents one mouse, n = 4. **e**, **f** Fbxw7 mRNA (**e**, n = 3) and protein (**f**) expression in Hepa 1-6 cells treated with DMSO or 5 μM C29 for 24 h. Data are represented as means ± SEM, *p < 0.05, unpaired two-tailed Student's t test (**c**, **e**). Source data are provided as a Source Data file.
