# Supplementary material for: Hepatocyte FBXW7-dependent activity of nutrient-sensing nuclear receptors controls systemic energy homeostasis and NASH progression in male mice
Source: Nat Commun. 2023 Nov 1;14:6982. doi: 10.1038/s41467-023-42785-3 (PMC10620240; doi:10.1038/s41467-023-42785-3)
Supplement: Supplementary file 3 — Description of Additional Supplementary Files [file 41467_2023_42785_MOESM3_ESM.docx]

**Description of Supplementary Data Files**

**Supplementary Data 1:** Hepatic FA compositions and metabolites of *Flox* and *Fbxw7^L-/-^* mice. Raw area counts were normalized to protein abundance in a parallel set of replicate samples and rescaled to set the average *Flox* value to 1. Missing values were not imputed. Any significantly upregulated and downregulated FAs and metabolites in *Fbxw7^L-/-^* liver are highlighted in red and blue, respectively (p < 0.05, unpaired two-tailed Student’s t test).

**Supplementary Data 2:** Gene expression profile of PPARα targets in FA metabolism found dysregulated in Fbxw7-null livers. Subset of mouse Hallmark FA metabolism gene signature genes (https://www.gsea-msigdb.org/) targeted by PPARa^1^ that are differentially expressed in *Fbxw7^L-/-^* vs *Flox* liver by re-analysis of RNA-seq data (n = 3, p < 0.05, FC > 1.30, Limma)^2^.

**Supplementary Data 3:** Liver ERRα ChIP-seq of *Flox* mice fasted for 24 h. ChIP-seq peaks were annotated to mouse reference genome mm10.

**Supplementary Data 4:** Liver ERRα ChIP-seq of *Fbxw7^L-/-^* mice fasted for 24 h. ChIP-seq peaks were annotated to mouse reference genome mm10.

**Supplementary Data 5:** Hepatic FA compositions of C29-treated *Fbxw7^L-/-^* mice. Mice were administered 15 mg/kg C29 solution by intraperitoneal injection for 14 days or vehicle solution as control. Control solution consisted of DMSO diluted (1:25) in 30% PEG-300 + 70% saline solution. Raw area counts were normalized to protein abundance in a parallel set of replicate samples and rescaled to set the average vehicle-treated *Fbxw7^L-/-^* value to 1. Missing values were not imputed. Any significantly upregulated and downregulated FAs and metabolites in C29-treated *Fbxw7^L-/-^* liver are highlighted in red and blue, respectively (p < 0.05, unpaired two-tailed Student’s t test).

**Supplementary Data 6:** Primer sets used in this study. Primer sets used for mouse genotyping, mouse ChIP-qPCR analysis as well as mouse and human RT-qPCR analyses are provided.

**Supplementary Data 7:** Antibody list. Antibodies used for immunoblotting, immunoprecipitation (IP) and chromatin immunoprecipitation (ChIP) are listed.

**Supplementary Data 8:** CoA MRM and MS Parameters. Optimized multiple reaction monitoring (MRM) transitions and retention time windows for acyl-CoA determination by LC-MS/MS.

Supplementary References

1. Lee, J. M. *et al*. Nutrient-sensing nuclear receptors coordinate autophagy. *Nature* **516**, 112-115, doi:10.1038/nature13961 (2014).

2. Zhao, X. *et al*. Circadian amplitude regulation via FBXW7-targeted REV-ERBα degradation. *Cell* **165**, 1644-1657, doi:10.1016/j.cell.2016.05.012 (2016).
