# Supplementary material for: Hepatocyte FBXW7-dependent activity of nutrient-sensing nuclear receptors controls systemic energy homeostasis and NASH progression in male mice
Source: Nat Commun. 2023 Nov 1;14:6982. doi: 10.1038/s41467-023-42785-3 (PMC10620240; doi:10.1038/s41467-023-42785-3)
Supplement: Supplementary file 12 — Reporting Summary [file 41467_2023_42785_MOESM12_ESM.pdf]

nature portfolio

Corresponding author(s): Vincent Giguère

Last updated by author(s): Oct 3, 2023

Reporting Summary

Nature Portfolio wishes to improve the reproducibility of the work that we publish. This form provides structure for consistency and transparency in reporting. For further information on Nature Portfolio policies, see our Editorial Policies and the Editorial Policy Checklist.

Statistics

For all statistical analyses, confirm that the following items are present in the figure legend, table legend, main text, or Methods section.

n/a	Confirmed	
☐	☒	The exact sample size (n) for each experimental group/condition, given as a discrete number and unit of measurement
☐	☒	A statement on whether measurements were taken from distinct samples or whether the same sample was measured repeatedly
☐	☒	The statistical test(s) used AND whether they are one- or two-sided<br>*Only common tests should be described solely by name; describe more complex techniques in the Methods section.*
☐	☒	A description of all covariates tested
☐	☒	A description of any assumptions or corrections, such as tests of normality and adjustment for multiple comparisons
☐	☒	A full description of the statistical parameters including central tendency (e.g. means) or other basic estimates (e.g. regression coefficient) AND variation (e.g. standard deviation) or associated estimates of uncertainty (e.g. confidence intervals)
☐	☒	For null hypothesis testing, the test statistic (e.g. F, t, r) with confidence intervals, effect sizes, degrees of freedom and P value noted<br>*Give P values as exact values whenever suitable.*
☒	☐	For Bayesian analysis, information on the choice of priors and Markov chain Monte Carlo settings
☒	☐	For hierarchical and complex designs, identification of the appropriate level for tests and full reporting of outcomes
☒	☐	Estimates of effect sizes (e.g. Cohen's d, Pearson's r), indicating how they were calculated

Our web collection on statistics for biologists contains articles on many of the points above.

Software and code

Policy information about availability of computer code

Data collection	1: Western blot: ChemiDoc MP imaging System (Bio-Rad).<br>2: Real-Time PCR: LightCycler 480 instrument (Roche).<br>3: Indirect calorimetry: Sable Systems International, Promethion high-definition behavioural phenotyping system.<br>4: Histology staining images: Aperio Scanscope XT (Leica Biosystems).<br>5: Metabolite profiling: LC-MS/MS using a triple quadrupole mass spectrometer (QQQ 6470) equipped with a 1290 ultra high-pressure liquid chromatography system (Agilent Technologies, Santa Clara, California, USA) and GC-MS using an Agilent 5975C GC/MS equipped with a DB-5MS+DG (30 m x 250 µm x 0.25 µm) capillary column or a Select FAMES (100 m x 250 µm x 0.25 µm) capillary column (Agilent J&W, Santa Clara, CA, USA).
Data analysis	1: Immunoblots presentation and quantification: Adobe photoshop 2021, Adobe illustrator 2021, and ImageJ (with Java 1.8.0_172).<br>2. RNA-seq analysis: The STAR software (version 2.5), DESeq2 R package (version 2_1.6.3), and R package limma. Phantasus (version 1.17.4) for PCA plot.<br>3: Transcriptome functional analysis: Enrichr and GSEA (version 4.2.3) with MSigDB (version 7.5.1).<br>4. Immune fraction score based on gene expression: CIBERSORT.<br>5. Estimation of cell types based on gene expression: EPIC.<br>6. Gene expression analysis of clinical samples: TNMplot and Kaplan–Meier Plotter.<br>7: Indirect calorimetry data analysis and graphing: CalR (version 1.3).<br>8: Histology staining images: Aperio ImageScope (version 12.4.3.5008) (Leica Biosystems).<br>9. Metabolomics analysis: MassHunter Quant (Agilent Technologies).<br>10. ChIP-seq analysis: Trimmomatic (version 0.36), BWA-MEM (version 0.7.12), Picard tools (version 2.0.1), MACS2 software suite (version

2.1.1.20160309), HOMER software suite (version 4.11.1), bedtools merge (version v2.27.0), deepTools (version 3.5.0) and IGV (version v2.8.6).
11: Statistics and graphing: GraphPad Prism 9 and Microsoft Excel (version 16.16.27).

For manuscripts utilizing custom algorithms or software that are central to the research but not yet described in published literature, software must be made available to editors and reviewers. We strongly encourage code deposition in a community repository (e.g. GitHub). See the Nature Portfolio guidelines for submitting code & software for further information.

Data

Policy information about availability of data

All manuscripts must include a data availability statement. This statement should provide the following information, where applicable:

- Accession codes, unique identifiers, or web links for publicly available datasets
- A description of any restrictions on data availability
- For clinical datasets or third party data, please ensure that the statement adheres to our policy

• ERRα ChIP-sequencing data performed on livers from Flox and Fbxw7L-/- male mice as well as liver RNA-sequencing data of WT and ERRα KO male mice fed a chow or a HFD for 15 weeks have been deposited in NCBI's Gene Expression Omnibus (GEO) and are accessible through GEO Series accession number GSE205847 encompassing SubSeries GSE205845 (ChIP-seq) and GSE205846 (RNA-seq). Raw RNA-seq data of Fbxw7-null and Flox livers (ZT1) were obtained from NCBI Sequence Read Archive (SRA ID: SRP059440). Raw PPARα liver ChIP-seq data of GW7647-treated WT or PPARα KO male mice as control were obtained from NCBI Sequence Read Archive (SRA ID: SRP047534). Reference mouse genome mm10 downloaded from the genome website browser (http://hgdownload.cse.ucsc.edu/goldenpath/mm10/bigZips/) was used for data analysis. Human NAFL/NASH and fibrotic liver biopsies RNA-seq GEO: GSE135251; cirrhotic human liver microarray GEO: GSE6764; gene expression in a panel of 12 mouse tissues GEO: GSE54650; fasted mouse liver RNA-seq GEO: GSE46495; hepatic profile post a keto diet GEO: GSE7699; and ERRα mouse liver ChIP-seq data GEO: GSE43638 used in this study were downloaded from GEO. Any additional information required to reanalyze the data reported in this paper is available from the lead contact upon request.
• This paper does not report original code.
• Source data values as well as uncropped immunoblots and gels are provided with this paper as Source Data files. Any additional information required to reanalyze the data reported in this paper is available from the lead contact upon request.

Human research participants

Policy information about studies involving human research participants and Sex and Gender in Research.

Reporting on sex and gender	*Use the terms sex (biological attribute) and gender (shaped by social and cultural circumstances) carefully in order to avoid confusing both terms. Indicate if findings apply to only one sex or gender; describe whether sex and gender were considered in study design whether sex and/or gender was determined based on self-reporting or assigned and methods used. Provide in the source data disaggregated sex and gender data where this information has been collected, and consent has been obtained for sharing of individual-level data; provide overall numbers in this Reporting Summary. Please state if this information has not been collected. Report sex- and gender-based analyses where performed, justify reasons for lack of sex- and gender-based analysis.*
Population characteristics	*Describe the covariate-relevant population characteristics of the human research participants (e.g. age, genotypic information, past and current diagnosis and treatment categories). If you filled out the behavioural & social sciences study design questions and have nothing to add here, write "See above."*
Recruitment	*Describe how participants were recruited. Outline any potential self-selection bias or other biases that may be present and how these are likely to impact results.*
Ethics oversight	*Identify the organization(s) that approved the study protocol.*

Note that full information on the approval of the study protocol must also be provided in the manuscript.

Field-specific reporting

Please select the one below that is the best fit for your research. If you are not sure, read the appropriate sections before making your selection.

☒ Life sciences ☐ Behavioural & social sciences ☐ Ecological, evolutionary & environmental sciences

For a reference copy of the document with all sections, see nature.com/documents/nr-reporting-summary-flat.pdf

Life sciences study design

All studies must disclose on these points even when the disclosure is negative.

Sample size	We did not compute statistical analyses to predetermine sample sizes before performing experiments. Sample sizes for each experiment are indicated.
Data exclusions	N/A
Replication	Experimental design was based on the use of at least 3 biological replicates as described in the figure legends and methods section generating

	consistent data as indicated by the significance of the results unless otherwise indicated.
Randomization	For in vivo study, mice analyzed were age matched, littermates and weight matched whenever possible, and they were then randomly allocated to each experimental group. For in vitro experiments, groups were allocated based on the genetic background of cells and different pharmacological treatments, thus no randomization was required. Randomization is not applicable for analyzing public dataset as groups have already been determined.
Blinding	Investigators were not blinded to experimental conditions for planning of experiments due to the complexity of the experiments, e.g. western blots require samples to be loaded in appropriate orders and mice in different genotypes were measured in the rotation manner to avoid the effects caused by difference in time. Data collection on indirect calorimetry is automated and confers high objectivity. The investigators were not blinded to group allocation during analysis of indirect calorimetry data, because software CalR (Version 1.3) was used for automatic quantification and unbiased comparison of differences between genotypes.

Reporting for specific materials, systems and methods

We require information from authors about some types of materials, experimental systems and methods used in many studies. Here, indicate whether each material, system or method listed is relevant to your study. If you are not sure if a list item applies to your research, read the appropriate section before selecting a response.

Materials & experimental systems

n/a	Involved in the study
☐	☒ Antibodies
☐	☒ Eukaryotic cell lines
☒	☐ Palaeontology and archaeology
☐	☒ Animals and other organisms
☐	☒ Clinical data
☒	☐ Dual use research of concern

Methods

n/a	Involved in the study
☐	☒ ChIP-seq
☒	☐ Flow cytometry
☒	☐ MRI-based neuroimaging

Antibodies

Antibodies used

ACADL:ABclonal;Cat#A1266
ACADM:St John's Laboratory;Cat#STJ96389
Acetyl-CoA Carboxylase:Cell Signaling Technology;Cat#3662S
ACOX1:ABclonal;Cat#A8091
Aif-1 (Iba-1):FUJIFILM Wako Pure Chemical Corporation;Cat#019-19741
αSMA:Thermo Fisher Scientific;Cat#14-9760-82
alpha-Tubulin:Cedarlane;Cat#CLT9002
AMPKα:Cell Signaling Technology;Cat#2532S
APCS:Proteintech;Cat#20773-1-AP
APOA4:Proteintech;Cat#17996-1-AP
Atg3:Cell Signaling Technology;Cat#3415S
Atg5:Cell Signaling Technology;Cat#12994S
Atg7:Cell Signaling Technology;Cat#2631S
ATGL:ABclonal;Cat#A6245
beta Actin:Abcam;Cat#ab8226
BNIP3:Abcam;Cat#ab10433
Caspase-12:Cell Signaling Technology;Cat#35965S
Caspase-3:Cell Signaling Technology;Cat#9662S
Caspase-9:Cell Signaling Technology;Cat#9504T
CHOP:Cell Signaling Technology;Cat#5554S
CHREBP:Novus Biologicals;Cat#NB400-135
Citrate synthetase:Abcam;Cat#ab96600
Cleaved PARP (Asp214):Cell Signaling Technology;Cat#9544S
Col1a1:Santa Cruz Biotechnology;Cat#sc-293182
COX7A2:ABclonal;Cat#A8406
CPT1A:ABclonal;Cat#A5307
CTGF:Santa Cruz Biotechnology;Cat#sc-365970
Cytochrome C:ABclonal;Cat#A13430
DECR1:Epitomics;Cat#S2218
Drp1:BD Transduction Laboratories;Cat#611113
eIF2α:Cell Signaling Technology;Cat#9722S
ERRα:Abcam;Cat#ab76228
Fatty Acid Synthase (FASN):ABclonal;Cat#A0461
FBXW7:R&D Systems;Cat#MAB7776
FBXW7:Bethyl Laboratories;Cat#A301-721A

Fis1:Santa Cruz Biotechnology;Cat#sc-98900
FoxO1:Cell Signaling Technology;Cat#2880T
GABARAPL1:Abcam;Cat#ab86497
GDF15:Proteintech;Cat#27455-1-AP
GLUT-4:Sigma-Aldrich;Cat#07-1404
Glycogen Synthase:Cell Signaling Technology;Cat#3886S
HSD17B4:St John's Laboratory;Cat#STJ96751
HSL:Cell Signaling Technology;Cat#4107S
IGFBP1:Cell Signaling Technology;Cat#31025S
JNK:Cell Signaling Technology;Cat#9252T
Lamin B1:Cell Signaling Technology;Cat#12586S
LAMP2:Abcam;Cat#ab13524
LC3B:Sigma-Aldrich;Cat#L7543
LDHA:Cell Signaling Technology;Cat#2012S
Lipin 1:Abcam;Cat#ab70138
MFF:Proteintech;Cat#17090-1-AP
Mfn2:Sigma-Aldrich;Cat#M6319
mTOR:Cell Signaling Technology;Cat#2983S
NDUFB3:ABclonal;Cat#A14378
OPA1:BD Transduction Laboratories;Cat#612607
p62/SQSTM1:Novus Biologicals;Cat#H00008878-M01
Parkin:Santa Cruz Biotechnology;Cat#sc-32282
PDK4:ABclonal;Cat#A13337
Perilipin-1:Cell Signaling Technology;Cat#9349S
Phospho-Acetyl-CoA Carboxylase (Ser79):Cell Signaling Technology;Cat#3661S
Phospho-AMPKα (Thr172):Cell Signaling Technology;Cat#2535S
Phospho-eIF2α (Ser51):Cell Signaling Technology;Cat#9721S
Phospho-Glycogen Synthase (Ser641):Cell Signaling Technology;Cat#47043T
Phospho-HSL (Ser563):Cell Signaling Technology;Cat#4139S
Phospho-HSL (Ser660):Cell Signaling Technology;Cat#4126S
Phospho-JNK (Thr183/Tyr185):Cell Signaling Technology;Cat#4668T
Phospho-mTOR (Ser2448):Cell Signaling Technology;Cat#2971S
phospho-p70 S6 Kinase (Thr389):Sigma-Aldrich;Cat#07-018-I
Phospho-ULK1 (Ser757):Cell Signaling Technology;Cat#6888S
PINK1:Santa Cruz Biotechnology;Cat#sc-517353
PPARα:Santa Cruz Biotechnology;Cat#sc-9000
PPARα:Millipore;Cat#MAB3890
S6K1:Abcam;Cat#ab32529
SREBP-1:Santa Cruz Biotechnology;Cat#8984X
TGF beta 2:Proteintech;Cat#19999-1-AP
Total OXPHOS Rodent WB antibody Cocktail:Abcam;Cat#ab110413
Ubiquitin:Cell Signaling Technology;Cat#3933S
ULK1:Cell Signaling Technology;Cat#8054S
UQCRQ:ABclonal;Cat#A9872
V5 tag:Abcam;Cat#ab9116
Vinculin:Sigma-Aldrich;Cat#MAB3574

Validation

ACADL:ABclonal;Cat#A1266;Manufacturer's website:https://abclonal.com/catalog-antibodies/ACADLPolyclonalAntibody/A1266
ACADM:St John's Laboratory;Cat#STJ96389;Manufacturer's website:https://stjohnslabs.com/anti-acadm-antibody-internal-stj96389/
Acetyl-CoA Carboxylase:Cell Signaling Technology;Cat#3662S;Manufacturer's website:https://www.cellsignal.com/products/primary-antibodies/acetyl-coa-carboxylase-antibody/3662
ACOX1:ABclonal;Cat#A8091;Manufacturer's website:https://abclonal.com/catalog-antibodies/ACOX1PolyclonalAntibody/A8091
Aif-1 (Iba-1):FUJIFILM Wako Pure Chemical Corporation;Cat#019-19741;Manufacturer's website:https://www.fujifilmcdi.com/anti-iba1-polyclonal-antibody-019-19741
αSMA:Thermo Fisher Scientific;Cat#14-9760-82;Manufacturer's website:https://www.thermofisher.com/antibody/product/Alpha-Smooth-Muscle-Actin-Antibody-clone-1A4-Monoclonal/14-9760-82
alpha-Tubulin:Cedarlane;Cat#CLT9002;Manufacturer's website:https://www.biocompare.com/9776-Antibodies/91113-Mouse-AntiHuman-BetaTubulin-Monoclonal-antibody-Unconjugated-Clone-dm1a/
AMPKα:Cell Signaling Technology;Cat#2532S;Manufacturer's website:https://www.cellsignal.com/products/primary-antibodies/ampka-antibody/2532?Ntk=Products&Ntt=2532
APCS:Proteintech;Cat#20773-1-AP;Manufacturer's website:https://www.ptglab.com/products/APCS-Antibody-20773-1-AP.htm
APOA4:Proteintech;Cat#17996-1-AP;Manufacturer's website:https://www.ptglab.com/products/APOA4-Antibody-17996-1-AP.htm
Atg3:Cell Signaling Technology;Cat#3415S;Manufacturer's website:https://www.cellsignal.com/products/primary-antibodies/atg3-antibody/3415
Atg5:Cell Signaling Technology;Cat#12994S;Manufacturer's website:https://www.cellsignal.com/products/primary-antibodies/atg5-d5f5u-rabbit-mab/12994
Atg7:Cell Signaling Technology;Cat#2631S;Manufacturer's website:https://www.cellsignal.com/products/primary-antibodies/atg7-antibody/2631?site-search-type=Products&N=4294956287&Ntt=atg7+2631&fromPage=plp&_requestid=1637934
ATGL:ABclonal;Cat#A6245;Manufacturer's website:https://abclonal.com/catalog-antibodies/ATGLPNPLA2RabbitpAb/A6245

beta Actin:Abcam;Cat#ab8226;Manufacturer's website:https://www.abcam.com/beta-actin-antibody-mabcam-8226-loading-control-ab8226.html
BNIP3:Abcam;Cat#ab10433;Manufacturer's website:https://www.abcam.com/bnip3-antibody-ana40-ab10433.html
Caspase-12:Cell Signaling Technology;Cat#35965S;Manufacturer's website:https://www.cellsignal.com/products/primary-antibodies/caspase-12-antibody/35965
Caspase-3:Cell Signaling Technology;Cat#9662S;Manufacturer's website:https://www.cellsignal.com/products/primary-antibodies/caspase-3-antibody/9662
Caspase-9:Cell Signaling Technology;Cat#9504T;Manufacturer's website:https://www.cellsignal.com/products/primary-antibodies/caspase-9-antibody-mouse-specific/9504
CHOP:Cell Signaling Technology;Cat#5554S;Manufacturer's website:https://www.cellsignal.com/products/primary-antibodies/chop-d46f1-rabbit-mab/5554
CHREBP:Novus Biologicals;Cat#NB400-135;Manufacturer's website:https://www.novusbio.com/products/chrebp-antibody_nb400-135
Citrate synthetase:Abcam;Cat#ab96600;Manufacturer's website:https://www.abcam.com/citrate-synthetase-antibody-ab96600.html
Cleaved PARP (Asp214):Cell Signaling Technology;Cat#9544S;Manufacturer's website:https://www.cellsignal.com/products/primary-antibodies/cleaved-parp-asp214-antibody-mouse-specific/9544
Col1a1:Santa Cruz Biotechnology;Cat#sc-293182;Manufacturer's website:https://www.scbt.com/p/col1a1-antibody-3g3
COX7A2:ABclonal;Cat#A8406;Manufacturer's website:https://abclonal.com/catalog-antibodies/COX7A2PolyclonalAntibody/A8406
CPT1A:ABclonal;Cat#A5307;Manufacturer's website:https://abclonal.com/catalog-antibodies/CPT1ARabbitpAb/A5307
CTGF:Santa Cruz Biotechnology;Cat#sc-365970;Manufacturer's website:https://www.scbt.com/p/ctgf-antibody-e-5?gclid=Cj0KCQiA4b2MBhD2ARIsAIrcB-RsbV3tU1zIEP2581W1P5QOdTjb_FsqLwt7jq5xXzw633ZxCqnj8SkaAuE_EALw_wcB
Cytochrome C:ABclonal;Cat#A13430;Manufacturer's website:https://abclonal.com/catalog-antibodies/CytochromecRabbitpAb/A13430
DECR1:Epitomics;Cat#S2218;Manufacturer's website:https://www.antibodypedia.com/gene/12737/DECR1/antibody/201195/S2218
Drp1:BD Transduction Laboratories;Cat#611113;Manufacturer's website:https://www.bdbiosciences.com/en-us/products/reagents/microscopy-imaging-reagents/immunofluorescence-reagents/purified-mouse-anti-dlp1.611113
eIF2α:Cell Signaling Technology;Cat#9722S;Manufacturer's website:https://www.cellsignal.com/products/primary-antibodies/eif2a-antibody/9722?site-search-type=Products&N=4294956287&Ntt=elf2a+9722s&fromPage=plp&_requestid=429302
ERRα:Abcam;Cat#ab76228;Manufacturer's website:https://www.abcam.com/estrogen-related-receptor-alpha-antibody-epr46y-ab76228.html
Fatty Acid Synthase (FASN):ABclonal;Cat#A0461;Manufacturer's website:https://abclonal.com/catalog-antibodies/FattyAcidSynthaseRabbitpAb/A0461
FBXW7:R&D Systems;Cat#MAB7776;Manufacturer's website:https://www.rndsystems.com/products/human-fbxw7-cdc4-antibody-800201_mab7776
FBXW7:Bethyl Laboratories;Cat#A301-721A;Manufacturer's website:https://www.thermofisher.com/antibody/product/FBW7-Antibody-Polyclonal/A301-721A
Fis1:Santa Cruz Biotechnology;Cat#sc-98900;Manufacturer's website:https://datasheets.scbt.com/sc-98900.pdf
FoxO1:Cell Signaling Technology;Cat#2880T;Manufacturer's website:https://www.cellsignal.com/products/primary-antibodies/foxo1-c29h4-rabbit-mab/2880
GABARAPL1:Abcam;Cat#ab86497;Manufacturer's website:https://www.abcam.com/gabarapl1-antibody-ab86497.html
GDF15:Proteintech;Cat#27455-1-AP;Manufacturer's website:https://www.ptglab.com/products/GDF15-Antibody-27455-1-AP.htm#publications
GLUT-4:Sigma-Aldrich;Cat#07-1404;Manufacturer's website:https://www.sigmaaldrich.com/CA/en/product/mm/071404
Glycogen Synthase:Cell Signaling Technology;Cat#3886S;Manufacturer's website:https://www.cellsignal.com/products/primary-antibodies/glycogen-synthase-15b1-rabbit-mab/3886
HSD17B4:St John's Laboratory;Cat#STJ96751;Manufacturer's website:https://stjohnslabs.com/anti-hsd17b4-antibody-n-term-stj96751/
HSL:Cell Signaling Technology;Cat#4107S;Manufacturer's website:https://www.cellsignal.com/products/primary-antibodies/hsl-antibody/4107
IGFBP1:Cell Signaling Technology;Cat#31025S;Manufacturer's website:https://www.cellsignal.com/products/primary-antibodies/igfbp1-d4e9t-xp-rabbit-mab/31025
JNK:Cell Signaling Technology;Cat#9252T;Manufacturer's website:https://www.cellsignal.com/products/primary-antibodies/sapk-jnk-antibody/9252?site-search-type=Products&Ns=productCitationsCount%7C1&N=4294956287&Ntt=jnk&fromPage=plp
Lamin B1:Cell Signaling Technology;Cat#12586S;Manufacturer's website:https://www.cellsignal.com/products/primary-antibodies/lamin-b1-d4q4z-rabbit-mab/12586?utm_strategy=lev&utm_conv=mon&utm_stage=ous&utm_tactic=ppc&utm_region=hq&gclid=Cj0KCQiAkuP9BRCkARIsAKGLE8VtvgMbQUUx28nkmNiS3y1igcNtGp-MdcWDxeqkXjLqkZdygjjqZrAaAkKGEALw_wcB&gclsrc=aw.ds
LAMP2:Abcam;Cat#ab13524;Manufacturer's website:https://www.abcam.com/lamp2-antibody-gl2a7-ab13524.html
LC3B:Sigma-Aldrich;Cat#L7543;Manufacturer's website:https://www.sigmaaldrich.com/CA/en/product/sigma/l7543
LDHA:Cell Signaling Technology;Cat#2012S;Manufacturer's website:https://www.cellsignal.com/products/primary-antibodies/ldha-antibody/2012
Lipin 1:Abcam;Cat#ab70138;Manufacturer's website:https://www.abcam.com/lipin-1-antibody-ab70138.html
MFF:Proteintech;Cat#17090-1-AP;Manufacturer's website:https://www.ptglab.com/products/MFF-Antibody-17090-1-AP.htm
Mfn2:Sigma-Aldrich;Cat#M6319;Manufacturer's website:https://www.sigmaaldrich.com/CA/en/product/sigma/m6319?gclid=Cj0KCQiA4b2MBhD2ARIsAIrcB-R8l-W_8EPk99MpeRbTCfH10KpiGiSPRmvGZzNfOEm_1Ugq3r5zdVAaAoecEALw_wcB
mTOR:Cell Signaling Technology;Cat#2983S;Manufacturer's website:https://www.cellsignal.com/products/primary-antibodies/mtor-7c10-rabbit-mab/2983
NDUFB3:ABclonal;Cat#A14378;Manufacturer's website:https://abclonal.com/catalog-antibodies/NDUFB3PolyclonalAntibody/A14378
OPA1:BD Transduction Laboratories;Cat#612607;Manufacturer's website:https://www.bdbiosciences.com/en-us/products/reagents/microscopy-imaging-reagents/immunofluorescence-reagents/purified-mouse-anti-opa1.612607
p62/SQSTM1:Novus Biologicals;Cat#H00008878-M01;Manufacturer's website:https://www.novusbio.com/products/p62-sqstm1-

antibody-2c11_h00008878-m01
Parkin:Santa Cruz Biotechnology;Cat#sc-32282;Manufacturer's website:https://www.scbt.com/p/parkin-antibody-prk8
PDK4:ABclonal;Cat#A13337;Manufacturer's website:https://abclonal.com/catalog-antibodies/PDK4PolyclonalAntibody/A13337
Perilipin-1:Cell Signaling Technology;Cat#9349S;Manufacturer's website:https://www.cellsignal.com/products/primary-antibodies/perilipin-1-d1d8-xp-rabbit-mab/9349
Phospho-Acetyl-CoA Carboxylase (Ser79):Cell Signaling Technology;Cat#3661S;Manufacturer's website:https://www.cellsignal.com/products/primary-antibodies/phospho-acetyl-coa-carboxylase-ser79-antibody/3661
Phospho-AMPKα (Thr172):Cell Signaling Technology;Cat#2535S;Manufacturer's website:https://www.cellsignal.com/products/primary-antibodies/phospho-ampka-thr172-40h9-rabbit-mab/2535?_=1604894915134&Ntt=2535S&tahead=true
Phospho-eIF2α (Ser51):Cell Signaling Technology;Cat#9721S;Manufacturer's website:https://www.cellsignal.com/products/primary-antibodies/phospho-eif2a-ser51-antibody/9721
Phospho-Glycogen Synthase (Ser641):Cell Signaling Technology;Cat#47043T;Manufacturer's website:https://www.cellsignal.com/products/primary-antibodies/phospho-glycogen-synthase-ser641-d4h1b-xp-rabbit-mab/47043?site-search-type=Products&N=4294956287&Ntt=+%28ser641%29+%28d4h1b%29+&fromPage=plp
Phospho-HSL (Ser563):Cell Signaling Technology;Cat#4139S;Manufacturer's website:https://www.cellsignal.com/products/primary-antibodies/phospho-hsl-ser563-antibody/4139
Phospho-HSL (Ser660):Cell Signaling Technology;Cat#4126S;Manufacturer's website:https://www.cellsignal.com/products/primary-antibodies/phospho-hsl-ser660-antibody/4126
Phospho-JNK (Thr183/Tyr185):Cell Signaling Technology;Cat#4668T;Manufacturer's website:https://www.cellsignal.com/products/primary-antibodies/phospho-sapk-jnk-thr183-tyr185-81e11-rabbit-mab/4668?site-search-type=Products&Ns=productCitationsCount%7C1&N=4294956287&Ntt=jnk&fromPage=plp
Phospho-mTOR (Ser2448):Cell Signaling Technology;Cat#2971S;Manufacturer's website:https://www.cellsignal.com/products/primary-antibodies/phospho-mtor-ser2448-antibody/2971
phospho-p70 S6 Kinase (Thr389):Sigma-Aldrich;Cat#07-018-I;Manufacturer's website:https://www.emdmillipore.com/CA/en/product/Anti-phospho-p70-S6-Kinase-Thr389-Antibody,MM_NF-07-018-I?ReferrerURL=https%3A%2F%2Fwww.google.com%2F
Phospho-ULK1 (Ser757):Cell Signaling Technology;Cat#6888S;Manufacturer's website:https://www.cellsignal.com/products/primary-antibodies/phospho-ulk1-ser757-antibody/6888
PINK1:Santa Cruz Biotechnology;Cat#sc-517353;Manufacturer's website:https://www.scbt.com/p/pink1-antibody-38ct20-8-5
PPARα:Santa Cruz Biotechnology;Cat#sc-9000;Manufacturer's website:https://datasheets.scbt.com/sc-9000.pdf
PPARα:Millipore;Cat#MAB3890;Manufacturer's website:https://www.emdmillipore.com/CA/en/product/Anti-PPAR-Antibody,MM_NF-MAB3890
S6K1:Abcam;Cat#ab32529;Manufacturer's website:https://www.abcam.com/s6k1-antibody-e343-ab32529.html
SREBP-1:Santa Cruz Biotechnology;Cat#8984X;Manufacturer's website:https://datasheets.scbt.com/sc-8984.pdf
TGF beta 2:Proteintech;Cat#19999-1-AP;Manufacturer's website:https://www.ptglab.com/products/TGFB2-Specific-Antibody-19999-1-AP.html
Total OXPHOS Rodent WB antibody Cocktail:Abcam;Cat#ab110413;Manufacturer's website:https://www.abcam.com/total-oxphos-rodent-wb-antibody-cocktail-ab110413.html
Ubiquitin:Cell Signaling Technology;Cat#3933S;Manufacturer's website:https://www.cellsignal.com/products/primary-antibodies/ubiquitin-antibody/3933
ULK1:Cell Signaling Technology;Cat#8054S;Manufacturer's website:https://www.cellsignal.com/products/primary-antibodies/ulk1-d8h5-rabbit-mab/8054
UQCRQ:ABclonal;Cat#A9872;Manufacturer's website:https://abclonal.com/catalog-antibodies/UQCRQRabbitpAb/A9872
V5 tag:Abcam;Cat#ab9116;Manufacturer's website:https://www.abcam.com/v5-tag-antibody-ab9116.html
Vinculin:Sigma-Aldrich;Cat#MAB3574;Manufacturer's website:https://www.emdmillipore.com/CA/en/product/Anti-Vinculin-clone-VIIF9-7F9,MM_NF-MAB3574-25UG

Eukaryotic cell lines

Policy information about cell lines and Sex and Gender in Research

Cell line source(s)	HepG2, HEK293T, and Hepa 1-6 cells were from the ATCC (Manassas, VA, USA).
Authentication	Authentication of HepG2 cells was based on the expression of insulin-like growth factor II.<br>HEK293T and Hepa 1-6 cells were not specifically authenticated beyond being obtained from ATCC. All cells were checked routinely for morphology and were used at a low passage to limit potential contamination.
Mycoplasma contamination	All cells utilized were periodically tested for mycoplasma contamination using a mycoplasma PCR detection kit (cat. no. G238; Applied Biological Materials) and showed no signs of infection.
Commonly misidentified lines (See ICLAC register)	No cell lines used are listed in the database of commonly misidentified cell lines.

Animals and other research organisms

Policy information about studies involving animals; ARRIVE guidelines recommended for reporting animal research, and Sex and Gender in Research

Laboratory animals	All mice experiments used age-matched male littermates (2- to 3-month-old), unless otherwise specified. Mice were housed two to five per cage at a constant environment (ambient temperature: 18°C-24°C; relative humidity: 30%-70%) under a 12-h light/dark cycle (7am-7pm light, 7pm-7am dark) with ad libitum access to water and a standard normal diet (ND; Envigo, Teklad Rodent diet 2920x; 3.1 kal/g, 24 kcal% protein, 16 kcal% fat, 60 kcal% carbohydrate) in an animal facility at McGill University. ERRα KO and ERRα

	phospho-mutant (3SA) mice on a C57BL/6N genetic background were described previously (PMID: 14585956 and PMID: 35440636). FBXW7 floxed mice (Stock No: 017563) on a C57BL/6J genetic background were obtained from the Jackson Laboratory and bred with Alb-Cre mice.
Wild animals	No wild animals were used in the study.
Reporting on sex	All mouse experimental data were derived from male mice.
Field-collected samples	No field collected samples were used in the study.
Ethics oversight	All mouse manipulations were performed in accordance with procedures approved by the McGill Facility Animal Care Committee within animal protocol 3173 and complied with ethical guidelines set by the Canadian Council of Animal Care.

Note that full information on the approval of the study protocol must also be provided in the manuscript.

Clinical data

Policy information about clinical studies

All manuscripts should comply with the ICMJEguidelines for publication of clinical research and a completedCONSORT checklist must be included with all submissions.

Clinical trial registration	*Provide the trial registration number from ClinicalTrials.gov or an equivalent agency.*
Study protocol	*Note where the full trial protocol can be accessed OR if not available, explain why.*
Data collection	*Describe the settings and locales of data collection, noting the time periods of recruitment and data collection.*
Outcomes	*Describe how you pre-defined primary and secondary outcome measures and how you assessed these measures.*

ChIP-seq

Data deposition

- [x] Confirm that both raw and final processed data have been deposited in a public database such as GEO.
- [x] Confirm that you have deposited or provided access to graph files (e.g. BED files) for the called peaks.

Data access links<br>*May remain private before publication.*	NCBI's Gene Expression Omnibus (GEO) SubSeries accession # GSE205845 part of GEO Series accession number # GSE205847.
Files in database submission	Raw files (Fastq, BAM) and processed files (annotated peaks, bigwig).
Genome browser session<br>(e.g. UCSC)	No longer available.

Methodology

Replicates	For each ChIP-seq, three ChIP replicates were pooled together prior to sequencing.
Sequencing depth	Flox_Liver_ERRα ChIP-seq: Total reads #112,171,410; Uniquely mapped reads (MAPQ > 20) #70,163,739; Sequenced as 100bp paired-end reads.<br>Fbxw7 LKO_ERRα ChIP-seq: Total reads #106,614,306; Uniquely mapped reads (MAPQ > 20) #67,339,449; Sequenced as 100bp paired-end reads.<br>Flox_Liver_input ChIP-seq: Total reads #90,434,890; Uniquely mapped reads (MAPQ > 20) #60,747,650; Sequenced as 100bp paired-end reads.<br>Fbxw7 LKO_input ChIP-seq: Total reads #94,098,086; Uniquely mapped reads (MAPQ > 20) #60,850,125; Sequenced as 100bp paired-end reads.
Antibodies	ERRα antibody (cat. no. ab76228; Abcam).
Peak calling parameters	ChIP-seq reads were first trimmed for adapter sequences and low-quality score bases using Trimmomatic v0.36. The resulting reads were mapped to the mouse reference genome (mm10) using BWA-MEM v0.7.12 in paired-end mode at default parameters. Only reads that had a unique alignment (mapping quality > 20) were retained and PCR duplicates were removed using Picard tools v2.0.1 (https://broadinstitute.github.io/picard/). Peaks were called using MACS2 software suite v2.1.1.20160309 at an FDR < 0.05 followed by subsequent filtering to FDR < 0.001 using respective sequenced libraries of input DNA as control.
Data quality	Given the large sequencing depth, peaks were first called using MACS2 software suite v2.1.1.20160309 at the default FDR cutoff of FDR < 0.05 using respective inputs as controls and peaks were further filtered to FDR < 0.001 (Flox_Liver_ERRα ChIP-seq: called peaks = 109,379; Fbxw7 LKO_ERRα ChIP-seq: called peaks = 204,399).
Software	Peak annotation and TF motif enrichment analysis were performed using the annotatePeaks and findMotifsGenome commands, respectively, from HOMER software suite v4.9.1. Separate "reference peak sets" was generated by merging ChIP-seq peaks across samples in the same experiment, using bedtools merge v2.27.0 with parameters: -sorted -d -150 (https://bedtools.readthedocs.io/). Peak signals (peak summit ± 150bp) were then calculated as Fragments Per Kilobase of transcript per Million mapped reads (FPKM)

using HOMER. ERRα and PPARα ChIP-seq tracks were visualized using IGV (version v2.8.6) and heatmaps were generated using “computeMatrix” followed by “plotHeatmap” from deepTools v3.5.0 to create binding intensity comparisons at peaks based on normalized (RPKM) bigwig track information.
